# Early-life risk factors for reversible and irreversible airflow limitation in young adults: findings from the BAMSE birth cohort

**DOI:** 10.1136/thoraxjnl-2020-215884

**Published:** 2020-11-12

**Authors:** Gang Wang, Inger Kull, Anna Bergström, Jenny Hallberg, Petra Um Bergström, Stefano Guerra, Goran Pershagen, Olena Gruzieva, Marianne van Hage, Antonios Georgelis, Christer Janson, Anders Lindén, Erik Melén

**Affiliations:** 1 Department of Integrated Traditional Chinese and Western Medicine, West China Hospital, Sichuan University, Chengdu, Sichuan, China; 2 Institute of Environmental Medicine, Karolinska Institutet, Stockholm, Sweden; 3 Department of Clinical Science and Education, Södersjukhuset, Karolinska Institutet, Stockholm, Sweden; 4 Sachs' Children's Hospital, Stockholm, Sweden; 5 Centre for Occupational and Environmental Medicine, Region Stockholm, Stockholm, Sweden; 6 Asthma and Airway Disease Research Center, The University of Arizona, Tucson, Arizona, USA; 7 ISGLOBAL, Barcelona, Spain; 8 Medicine Solna, Division of Immunology and Allergy, Karolinska Institutet, Stockholm, Sweden; 9 Department of Medical Sciences: Respiratory, Allergy and Sleep Research, Uppsala University, Uppsala, Sweden; 10 Department of Respiratory Medicine and Allergy, Karolinska University Hospital, Stockholm, Sweden

**Keywords:** COPD epidemiology

## Abstract

We aimed to determine prevalence and early-life risk factors for reversible and irreversible airflow limitation in young adults from the general population. Among young adults in their 20s, the prevalence was 5.3% for reversible airflow limitation and 2.0% for irreversible airflow limitation. While parental asthma was the only risk factor for development of reversible airflow limitation, the risk factors for development of irreversible airflow limitation were current asthma, childhood respiratory tract infections and asthma, and exposure to air pollution.

It has been shown that impaired lung function in children and young adults is associated with an increased risk of chronic obstructive pulmonary disease (COPD) later in life.^1 2^ Recently, airflow limitation defined as pre-bronchodilator (BD) forced expiratory volume in 1 s (FEV_1_)/forced vital capacity (FVC) below the lower limit of normal (LLN) was observed in 4% of adults aged 20–29 years who have less than 5 pack-years tobacco load, and up to 7% in participants who have 5 pack-years or more tobacco load.^3^ However, few published studies addressed reversible airflow limitation and irreversible airflow limitation in young adults. Given this, we aimed to determine the prevalence and early-life risk factors for reversible airflow limitation and irreversible airflow limitation in young adults from the general population.

A total of 1932 participants in the population-based birth cohort Barn/Children, Allergy, Milieu, Stockholm, Epidemiology (BAMSE) performed valid pre-BD and post-BD lung function measurements at the 24-year follow-up.^4 5^ Lung function was tested according to American Thoracic Society (ATS)/European Respiratory Society (ERS) criteria as previously described.^6^ Post-BD lung function was tested 15 min after administration of 400 µg salbutamol. ‘Normal lung function’ was defined as pre-BD and post-BD FEV_1_/FVC ≥LLN,^7^ ‘Reversible airflow limitation’ as pre-BD FEV_1_/FVC <LLN but post-BD FEV1/FVC≥LLN, and ‘Irreversible airflow limitation’ as pre-BD and post-BD FEV_1_/FVC <LLN. ORs and 95% CIs for risk factors in relation to reversible airflow limitation or irreversible airflow limitation, selected based on previous literature and availability in BAMSE, were estimated using multivariable logistic regression in R (V.4.0.2).

The prevalence of reversible airflow limitation was 5.3% (n=103, 95% CI 4.3% to 6.3%), and irreversible airflow limitation 2.0% (n=39, 95% CI 1.4% to 2.6%) at the 24-year follow-up. Forty-nine per cent reported respiratory symptoms in those with irreversible airflow limitation compared with 25% in those with normal lung function (table 1). In addition, participants in the irreversible airflow limitation group also reported more cough, but not more mucus production, during winter mornings. Besides, there were reports of more respiratory symptoms (defined as troublesome breasing, chest tightness or wheezing) and pneumonia events during the last 12 months in the irreversible airflow limitation group. However, no such differences were observed for the groups with reversible airflow limitation and normal lung function. There were lower pre-BD FEV_1_ and post-BD FEV_1_ (by design, online supplemental table E1), higher pre-BD FVC and post-BD FVC, and higher reversibility (change in FEV_1_ and change in FEV_1_ % baseline) in the reversible and irreversible airflow limitation groups compared with the group with normal lung function. Fifteen and 2.9% of participants with irreversible and reversible airflow limitation had post-BD FEV_1_ lower than LLN, respectively, compared with 1.5% in the group with normal lung function (online supplemental table E1). No substantial difference was observed for other variables, except higher body mass index (BMI) was observed in participants with irreversible airflow limitation (table 1). In logistic regression models adjusted for age, gender and BMI, several indicators of early life infections and respiratory diseases, environmental exposures and current asthma were risk factors associated with irreversible airflow limitation (table 2). In mutually adjusted analyses, respiratory syncytial virus (RSV) infection/pneumonia during infancy, nitrogen oxide (NO_x_) exposure during age 0–1 years, childhood asthma during age 0–4 years and current asthma were independent risk factors for irreversible airflow limitation (table 3). For reversible airflow limitation, parental asthma and childhood asthma during age 0–4 and 8–12 years, NO_x_ exposure during age 8–12 years and current asthma were associated factors. In mutually adjusted analyses, parental asthma alone was an independent risk factor for reversible airflow limitation.

10.1136/thoraxjnl-2020-215884.supp1 Supplementary data

**Table 1:** Characteristics of cohort participants with Irreversible airflow limitation or Reversible airflow limitation and normal lung function*

	Irreversible airflow limitation (N=39)	Reversible airflow limitation (N=103)	Normal lung function (N=1790)	P value	
				Irreversible airflow limitation versus normal lung function	Reversible airflow limitation versus normal lung function
Age, years, mean (SD)	22.4 (0.5)	22.4 (0.5)	22.4 (0.5)	0.7387	0.5593
Sex, female, n (%)	19 (48.7)	58 (56.3)	1042 (58.2)	0.2346	0.7037
BMI, kg/m^2^, mean (SD)	24.9 (5.9)	23.2 (3.0)	23.1 (3.8)	0.0060	0.9549
Education, n (%)				0.1506	0.7524
Secondary school	20 (51.3)	62 (60.2)	1118 (62.6)		
High school	9 (23.1)	23 (22.3)	405 (22.7)		
University	10 (25.6)	18 (17.5)	264 (14.8)		
Active childhood smoking, n (%)				0.2804	0.8711
Never	23 (59.0)	70 (68.0)	1200 (67.2)		
Ever	16 (41.0)	33 (32.0)	586 (32.8)		
Tobacco consumption (pack-years), median (IQR)	1.1 (0.1 to 1.6)	1.0 (0.1 to 2.3)	0.4 (0.1 to 1.7)	0.4504 **†**	0.3609 **†**
Respiratory health events					
Cough during winter morning, n (%)	9 (23.1)	11 (10.8)	139 (7.9)	0.0006	0.3686
Mucus production during winter morning, n (%)	6 (15.8)	18 (17.8)	257 (14.6)	0.8319	0.4999
Respiratory symptoms, n (%)	19 (48.7)	34 (33.0)	451 (25.2)	0.0009	0.0791
Pneumonia in the last 12 months, n (%)	4 (10.3)	3 (3.0)	44 (2.5)	0.0183‡	0.7387‡
Sensitisation at age 24 years					
Sensitisation to airborne allergens, n (%)	18 (46.2)	36 (35.6)	767 (43.5)	0.7414	0.1207
Sensitisation to food allergens, n (%)	5 (12.8)	10 (9.9)	153 (8.7)	0.3826‡	0.6736
Reversibility test					
Change in FEV_1_, ml, median (IQR)	243 (156 to 360)	273 (192 to 373)	111 (50 to 185)	<0.0001**†**	<0.0001**†**
Change in FEV_1_ % baseline, %, median (IQR)	7.3 (4.2 to 10.2)	7.7 (5.1 to 9.9)	2.8 (1.3 to 4.6)	<0.0001**†**	<0.0001**†**
Blood different cell count, ×10^9^/L					
Neutrophils, mean (SD)	3.86 (1.5)	3.51 (1.37)	3.71 (1.4)	0.5358	0.1734
Eosinophils, median (IQR)	0.10 (0.1 to 0.2)	0.10 (0.0 to 0.2)	0.10 (0.0 to 0.2)	0.0529**†**	0.7534**†**

*Details about the definitions of health outcomes and covariates are provided in the online supplemental file.

†Based on Kruskal-Wallis rank sum test.

‡Based on Fisher’s exact test.

BMI, body mass index; FEV_1_, forced expiratory volume in 1 s.

**Table 2:** Logistic regression model reversible and irreversible airflow limitation adjusted for age, gender, and body mass index*

Variables	Normal lung function		Reversible airflow limitation		Irreversible airflow limitation	
	No of individuals/cases	OR (95% CI)	No of individuals/cases	OR (95% CI)	No of individuals/cases	OR (95% CI)
Parental education, primary school/high school versus university	1787/774	1 (Reference)	102/40	0.85 (0.56 to 1.28)	39/17	0.98 (0.51 to 1.86)
Maternal smoking during pregnancy	1789/197	1 (Reference)	103/14	1.26 (0.70 to 2.27)	39/5	1.13 (0.44 to 2.96)
Parental asthma	1776/342	1 (Reference)	103/36	2.24 (1.47 to 3.42)	39/7	0.9 (0.39 to 2.07)
Preterm birth	1790/95	1 (Reference)	103/5	0.91 (0.36 to 2.28)	39/3	1.42 (0.43 to 4.71)
Parental smoking during childhood	1790/526	1 (Reference)	103/33	1.13 (0.74 to 1.73)	39/12	1.02 (0.51 to 2.05)
Active childhood smoking, ever versus never	1786/586	1 (Reference)	103/33	0.97 (0.63 to 1.48)	39/16	1.40 (0.73 to 2.69)
RSV infection/pneumonia during infancy	1745/125	1 (Reference)	99/3	0.40 (0.13 to 1.29)	39/9	3.72 (1.71 to 8.11)
Pneumonia during age 0–4 years	1772/189	1 (Reference)	100/7	0.62 (0.29 to 1.37)	39/9	2.42 (1.12 to 5.23)
Childhood asthma during age 0–4 years	1772/179	1 (Reference)	100/17	1.81 (1.04 to 3.13)	38/11	3.26 (1.57 to 7.78)
Childhood asthma during age 4–8 years	1606/99	1 (Reference)	91/10	1.86 (0.93 to 3.70)	35/4	1.73 (0.58 to 5.11)
Childhood asthma during age 8–12 years	1589/108	1 (Reference)	93/12	1.98 (1.04 to 3.76)	36/3	1.08 (0.32 to 3.64)
Childhood asthma during age 12–16 years	1560/104	1 (Reference)	94/11	1.84 (0.95 to 3.58)	36/4	1.58 (0.54 to 4.68)
Current asthma	1783/210	1 (Reference)	100/17	2.32 (1.43 to 3.77)	39/11	2.75 (1.34 to 5.66)
NO_x_ concentration higher than the median†						
Age 0–1 years	1767/911	1 (Reference)	103/45	0.72 (0.48 to 1.08)	39/28	2.61 (1.28 to 5.35)
Age 1–4 years	1640/859	1 (Reference)	88/39	0.72 (0.47 to 1.11)	37/24	1.74 (0.88 to 3.44)
Age 4–8 years	1488/742	1 (Reference)	81/38	0.88 (0.56 to 1.38)	34/21	1.71 (0.85 to 3.46)
Age 8–12 years	1637/832	1 (Reference)	90/36	0.64 (0.41 to 0.98)	37/22	1.53 (0.78 to 2.99)
Age 12–16 years	1453/737	1 (Reference)	82/38	0.82 (0.53 to 1.29)	33/22	2.19 (1.04 to 4.62)
PM_10_ concentration higher than the median‡						
Age 0–1 years	1767/914	1 (Reference)	103/47	0.78 (0.52 to 1.17)	39/29	2.96 (1.42 to 6.18)
Age 1–4 years	1640/840	1 (Reference)	88/43	0.89 (0.58 to 1.37)	37/26	2.39 (1.16 to 4.92)
Age 4–8 years	1488/759	1 (Reference)	81/39	0.91 (0.58 to 1.42)	34/22	1.79 (0.88 to 3.67)
Age 8–12 years	1637/827	1 (Reference)	90/36	0.65 (0.42 to 1.00)	37/22	1.54 (0.79 to 3.02)
Age 12–16 years	1453/742	1 (Reference)	82/36	0.76 (0.48 to 1.19)	33/21	1.85 (0.89 to 3.84)

*Details about the definitions of the covariates are provided in the online supplemental file.

†The median NO_x_ concentration for ages 0–1, 1–4, 4–8, 8–12 and 12–16 years were 29.16, 24.21, 18.31, 13.66 and 11.16 µg/m^3^.

‡The median PM_10_ concentration for ages 0–1, 1–4, 4–8, 8–12, and 12–16 years were 14.46, 13.28, 13.33, 13.34 and 12.24 µg/m^3^.

NO_x_, nitrogen oxides; PM_10_, particulate matter with aerodynamic diameter <10 µm; RSV, respiratory syncytial virus.

**Table 3:** Mutually adjusted analyses for reversible and irreversible airflow limitation which included age, gender, body mass index and other related factors*

Variables	Normal lung function		Irreversible airflow limitation	
	No of individuals/cases	OR (95% CI)	No of individuals/cases	OR (95% CI)
NO_x_ concentration during age 0–1 years†, high versus low	1261/651	1 (Reference)	29/22	3.08 (1.11 to 8.51)
NO_x_ concentration during age 12–16 years†, high versus low	1261/637	1 (Reference)	29/21	2.12 (0.82 to 5.46)
RSV infection/pneumonia during infancy	1261/84	1 (Reference)	29/8	3.59 (1.41 to 9.14)
Childhood asthma during age 0–4 years	1261/127	1 (Reference)	29/8	3.36 (1.22 to 9.24)
Childhood asthma during age 4–8 years	1261/78	1 (Reference)	29/2	0.32 (0.06 to 1.81)
Childhood asthma during age 8–12 years	1261/82	1 (Reference)	29/1	0.16 (0.02 to 1.43)
Childhood asthma during age 12–16 years	1261/78	1 (Reference)	29/3	1.31 (0.32 to 5.44)
Current asthma	1261/144	1 (Reference)	29/7	3.51 (1.22 to 10.15)
**Variables**	**Normal lung function**		**Reversible airflow limitation**	
Parental asthma	1317/253	1 (Reference)	77/28	2.17 (1.31 to 3.58)
NO_x_ concentration during age 8–12 years†, high versus low	1317/654	1 (Reference)	77/34	0.82 (0.51 to 1.32)
Childhood asthma during age 0–4 years	1317/137	1 (Reference)	77/14	1.37 (0.68 to 2.75)
Childhood asthma during age 4–8 years	1317/84	1 (Reference)	77/9	0.92 (0.36 to 2.34)
Childhood asthma during age 8–12 years	1317/88	1 (Reference)	77/10	1.22 (0.50 to 2.97)
Childhood asthma during age 12–16 years	1317/87	1 (Reference)	77/10	1.21 (0.51 to 2.86)
Current asthma	1317/154	1 (Reference)	77/16	1.49 (0.72 to 3.11)

*Details about the definitions of covariates are provided in the online supplemental file.

†The median NO_x_ concentration for ages 0–1, 8–12 and 12–16 years were 29.16, 13.66 and 11.16 µg/m^3^.

NO_x_, nitrogen oxides; RSV, respiratory syncytial virus.

In summary, we found the overall prevalence of reversible and irreversible airflow limitation to be rather high (5.3% and 2.0%, respectively), considering the young age of the participants. Individuals with irreversible airflow limitation more often reported current respiratory symptoms and pneumonia compared with those with normal lung function. Besides, more severe lung function impairments were observed in individuals with irreversible airflow limitation. Thus, there was a substantial disease burden in participants with irreversible airflow limitation. Results from previous epidemiology studies demonstrate that abnormal lung development plays an important role in the development of COPD and a substantial proportion of subjects diagnosed with COPD after age 50 could be traced back to a relatively low peak lung function in their 20s.^8^ Our current findings extend those results by demonstrating that irreversible airflow limitation is present in young adults. This was observed despite most of them being non-smokers, and ever-smokers having smoked on average less than one pack-year. Moreover, although only 28% of the irreversible airflow limitation subjects were classified as having current asthma, enhanced airway reversibility was observed, which suggests that, at this early stage of disease, reversibility is to some extent present.

In our study, early-life respiratory infections (RSV infection/pneumonia) and exposure to air pollutants, as well as childhood asthma, were identified as strong risk factors for irreversible airflow limitation. Given that air pollution levels in Stockholm are comparatively low by international standards, this makes the current findings alarming in a global context. Early risk factors may influence lung function development in a negative manner that will likely track with age,^9^ and in the current study, these early lung insults were even stronger risk factors for irreversible airflow limitation than smoking exposure in childhood and active smoking in adolescence. Thus, early-life exposure to air pollutants emerges as an important risk factor, known to be associated with not only lung development,^6^ but also with childhood pneumonia^10^ and asthma.^11^

In our study, the only identified independent risk factor for reversible airflow limitation was parental asthma, which suggests that the reversible airflow limitation phenotype relates primarily to asthma heredity, rather than to impaired lung development.

Our study has the drawback of a relatively small irreversible airflow limitation sample size and only around 50% of the initial 4089 cohort participants provided pre-BD and post-BD spirometry data at 24 years of age. However, no selection bias could be identified so far in the BAMSE cohort.^4^

In conclusion, this study forwards evidence that among young adults in their 20s, the prevalence was 5.3% for reversible airflow limitation and 2.0% for irreversible airflow limitation. While parental asthma emerges as the only risk factor for development of reversible airflow limitation, the risk factors for development of irreversible airflow limitation were current asthma, childhood respiratory tract infections and asthma, and exposure to air pollution.
