# Supplementary material for: Early-life risk factors for reversible and irreversible airflow limitation in young adults: findings from the BAMSE birth cohort
Source: Thorax. 2020 Nov 12;76(5):503–7. doi: 10.1136/thoraxjnl-2020-215884 (PMC8070638; doi:10.1136/thoraxjnl-2020-215884)
Supplement: Supplementary data [file thoraxjnl-2020-215884supp001.pdf]

Online Data Supplement

"Early-life risk factors for reversible and irreversible airflow limitation in young adults: Findings from the BAMSE birth cohort"

Authors:

Gang Wang, MD; Inger Kull, PhD; Anna Bergström, PhD; Jenny Hallberg, MD, PhD; Petra Um Bergström, MD, PhD; Stefano Guerra, MD, PhD; Göran Pershagen, MD, PhD; Olena Gruzieva, MD, PhD; Marianne van Hage, MD, PhD; Antonios Georgelis, PhD; Christer Janson, MD, PhD; Anders Lindén, MD, PhD; Erik Melén, MD, PhD

Text

Definitions of potential confounders and covariates

Tobacco consumption (pack-years) were calculated based on current smokers and assessed as multiplying the number of packs of cigarettes smoked per day by the number of years the person has smoked.

Respiratory symptoms were assessed as any troublesome breathing, chest tightness or wheezing during the last 12 months.

Sensitization was determined as a positive Phadiatop (a mix of common inhalant allergens) and/or fx5 (a mix of common food allergens) (specific IgE $\geq$ 0.35 kU_A/L).

Maternal smoking during pregnancy was defined as the mother smoked at least one cigarette per day at any point in time during pregnancy.

Parental asthma was defined as mother and/or father with self-reported diagnosis of asthma at the time of questionnaire 0.

Preterm birth was defined as the birth of a baby that occurs before the start of the 37th week of pregnancy.

Parental smoking during childhood was defined as any of the parents smoking $\geq$1 cigarette per day during age 0-16 years.

Respiratory syncytial virus infection/Pneumonia during infancy was defined as a doctor diagnosed with respiratory syncytial virus infection or pneumonia during age 0-1 years.

Childhood asthma during ages 0-4, 4-8, 8-12 and 12-16 years was defined if at least two of the following three criteria were fulfilled: doctor’s diagnosis of asthma ever; wheezing in the last 12 months; and/or use of asthma medication in the last 12 months at ages 1, 2 or 4 and 8, 12 or 16 years, respectively.

Current asthma was defined as a positive answer to doctor diagnosis of asthma, and at least one of the following: wheezing in the last 12 months; or use of asthma medication in the last 12 months.

Tables

Table 1. Pre- and post-bronchodilator lung function of cohort participants with reversible airflow limitation or irreversible airflow limitation and normal lung function.

	Irreversible airflow limitation (N=39)	Reversible airflow limitation (N=103)	Normal lung function (N=1790)	P value	
				Irreversible airflow limitation vs. Normal lung function	Reversible airflow limitation vs. Normal lung function
Spirometry data					
% predicted * pre-BD FEV_1, mean (SD)	83.4 (8.6)	88.7 (9.0)	97.6 (9.7)	< 0.0001	< 0.0001
Pre-BD FEV_1 z-score *, mean, n (%)	-1.42 (0.73)	-0.96 (0.77)	-0.20 (0.83)	< 0.0001	< 0.0001
% predicted * pre-BD FVC, mean (SD)	106.0 (9.9)	104.7 (10.3)	98.9 (10.2)	< 0.0001	< 0.0001
Pre-BD FVC z-score *, mean (SD)	0.50 (0.83)	0.38 (0.85)	-0.10 (0.84)	< 0.0001	< 0.0001
Pre-BD FEV_1/FVC, %, mean (SD)	66.9 (3.4)	72.1 (2.2)	84.4 (5.1)	< 0.0001	< 0.0001
Pre-BD FEV_1/FVC z-score *, mean (SD)	-2.44 (0.30)	-1.90 (0.20)	-0.23 (0.78)	< 0.0001	< 0.0001
% predicted * post-BD FEV_1, mean (SD)	90.1 (8.7)	95.4 (9.5)	100.4 (9.6)	< 0.0001	< 0.0001
Post-BD FEV_1 z-score *, mean (SD)	-0.85 (0.74)	-0.39 (0.81)	0.04 (0.83)	< 0.0001	< 0.0001

Post-BD FEV1 lower than LLN *, n (%)	6 (15.4%)	3 (2.9%)	27 (1.5%)	< 0.0001	0.2213 ll
% predicted * post-BD FVC, mean (SD)	106.3 (9.9)	104.3 (10.4)	98.4 (10.2)	< 0.0001	< 0.0001
Post-BD FVC z-score *, mean (SD)	0.51 (0.83)	0.35 (0.86)	-0.14 (0.85)	< 0.0001	< 0.0001
Post-BD FEV_1/FVC, %, mean (SD)	72.1 (2.1)	77.9 (2.7)	87.3 (4.6)	< 0.0001	< 0.0001
Post-BD FEV_1/FVC z-score *, mean (SD)	-1.88 (0.20)	-1.17 (0.30)	0.23 (0.74)	< 0.0001	< 0.0001

SD: standard deviation; BD: bronchodilator; FEV_1: forced expiratory volume in 1 second; LLN: lower limit of normal; FVC: forced vital capacity.

* Based on the reference equation from the Global Lung Initiative 2012 [1].

ll Based on Fisher's exact test.

References

1. Quanjer PH, Stanojevic S, Cole TJ, et al. Multi-ethnic reference values for spirometry for the 3-95-yr age range: the global lung function 2012 equations. *Eur Respir J* 2012;40(6):1324-43. doi: 10.1183/09031936.00080312
